# Effect of whole-plant mulberry supplementation on testis development and antioxidant capacity in Hu rams

**DOI:** 10.5713/ab.24.0627

**Published:** 2025-02-27

**Authors:** Jiamei Liu, Ziwei Liang, Wanhong Li, Xiuxiu Weng, Xiangpeng Yue, Fadi Li

**Affiliations:** 1 State Key Laboratory of Herbage Improvement and Grassland Agro-Ecosystems, Key Laboratory of Grassland Livestock Industry Innovation, Ministry of Agriculture and Rural Affairs, Engineering Research Center of Grassland Industry, Ministry of Education, College of Pastoral Agriculture Science and Technology, Lanzhou University, Lanzhou, China

**Keywords:** Antioxidation, Hu Sheep, Leydig Cell, Proanthocyanidins, Testis Development

## Abstract

**Objective:**

This study investigated the effects of dietary whole-plant mulberry (WM) on testicular development and antioxidant performance in sheep.

**Methods:**

Fifty-four three-month-old Hu sheep were divided into three groups and fed diets containing different proportions of WM (WM0, without WM; WM4, 4% WM; WM8, 8% WM). Following a 70-day feeding trial, 15 individuals from each group were humanely slaughtered. The total cholesterol (T-CHO) levels and antioxidant capacity of the testes were measured. The expression of functional genes was assessed by reverse transcription quantitative polymerase chain reaction. Leydig cells treated with proanthocyanidins (PCs) at concentrations of 0, 5, 10, and 20 μM. The total antioxidant capacity level (T-AOC), testosterone (T) level, cell viability, apoptosis and necrosis ratio were assessed. RNA sequencing analysis was performed to identify the differentially expressed genes (DEGs) between the control (CK) and 10 μM PC groups.

**Results:**

The PCs content in the WM was measured at 56.93±2.146 mg/g. The total (p = 0.06), the left (p = 0.07). and the right (p<0.05) testicular weights were increased in the WM8 group compared to the WM0 group. Compared to the WM0 group, the WM8 group showed decreased T-CHO (p<0.05) and increased T-AOC (p<0.05) in the testis, and the expression of *StAR*, *PPARγ* and *Bcl2* was significantly increased (p<0.05), while *Caspase9* and *Caspase3* were significantly decreased (p<0.05). *In vitro*, supplementation of 10 μM PCs in Leydig cells significantly increased cell viability, T-AOC and T levels, and reduced the necrosis ratio (p<0.05) compared to the CK group. Kyoto Encyclopedia of Genes and Genomes (KEGG) pathway enrichment analysis showed that the DEGs were significantly enriched in the steroid biosynthesis pathway, p53 signaling pathway, cholesterol metabolism, PPAR signaling pathway, and Hippo signaling pathway.

**Conclusion:**

Supplementation with 8% WM improved antioxidant capacity and stimulated testis development through the promotion of cell proliferation, T synthesis, and antioxidant capacity of Leydig cells.

## INTRODUCTION

Mulberry (*Morus alba* L.) possesses a strong tolerance to drought and saline–alkali environments, which can adapt to the ecological conditions of China’s northwest region, where it can help address desertification problems. Mulberry leaves have been fed as roughage in livestock and poultry to increase usage. Compared with other forages, mulberry leaves have high crude protein content (14.0% to 34.2%) and organic matter digestibility, rich in mineral elements and vitamins, which can optimize the nutritional value and considered as good feed ingredient for ruminants [1,2].

Mulberry contains various bioactive compounds that benefit animal health. The attention on bioactive substances in mulberry mainly centers on its functional substances such as antioxidants, anticancer agents, anti-inflammatory compounds, and lipid-lowering substances [3]. Due to differences in climate and environment across various regions, which result in different cutting times and methods, these factors affect the content of active substances in mulberry leaves. The content of bioactive substances in mulberry leaves varies greatly among varieties, with γ-aminobutyric acid ranging from 0.08 to 0.63 mg/g, total amino acids from 60.65 to 104.24 μg/g, total sugars from 29.36 to 73.37 mg/g, reducing sugars from 13.19 to 139.58 mg/g, flavonoids from 17.83 to 34.40 mg/g, total phenols from 5.68 to 16.78 mg/g, and total alkaloids from 4.29 to 9.82 mg/g [4]. The active chemicals contained in mulberry can serve as a source of natural antioxidants, and mulberry plants are particularly rich in flavonoid actives, which can be found in mulberry leaves at levels up to 34.10 to 68.32 mg/g [5]. Among these bioactive substances, proanthocyanidins (PCs) have strong antioxidant effects and can effectively scavenge superoxide anion radicals and hydroxyl radicals, which are internationally recognized as some of the most effective natural antioxidants for scavenging free radicals in the human body.

Reactive oxygen species (ROS), metabolic byproducts of aerobic cellular respiration neutralized by the antioxidant defense system, are widely studied for their detrimental effects on male fertility. ROS has been proved to be a causative molecule for spontaneous and inheritable mutations in the germ lineage cells. From spermatogonium differentiation to sperm maturation, the imbalances in the oxidant/antioxidant system can disrupt spermatogenesis. Various factors, such as infections, aging, obesity, and heat stress, can elevate ROS levels in rams. High concentrations of ROS can cause oxidative damage to DNA or unsaturated fatty acids, leading to the formation of 8-hydroxy, 2′-deoxyguanosine (8OHdG), malondialdehyde (MDA), and 4-hydroxynonenal. ROS induce DNA damage, including single or double strand breaks, base modifications, mitochondrial DNA damage, epigenetic alterations, and Y chromosomal microdeletions [6]. Polyunsaturated fatty acids (PUFAs), which are abundant in the sperm membrane, are particularly susceptible to oxidative damage, resulting in reduced sperm motility and fertility [7]. Recent research indicated that diets rich in bioactive compounds with antioxidant properties could promote testicular development and spermatogenesis. Additionally, the PUFAs composition of sperm membrane could be altered, and sperm motility could be improved by optimizing the dietary fatty acid proportion [8].

We speculate that the mulberry feed may have similar effects on alleviating oxidative stress in the testes given the antioxidant ability of its bioactive compounds. A total of 54 three-month-old Hu sheep were fed with different proportions of whole-plant mulberry (WM) for 70 days to explore the effect of WM as a feed material on the testicular development of Hu sheep. After that, *in vitro* experiments on Leydig cells were conducted to verify the effects of PCs on the cell proliferation, antioxidant capacity, and T synthesis.

## MATERIALS AND METHODS

### Measurement of proanthocyanidins content

The content of PCs in WM was determined using a detection kit obtained from Nanjing Jiancheng Bioengineering Institute (Nanjing, China). The WM was dried and then grounded into powder. The powder was sieved through a 40-mesh sieve, and approximately 0.02 g of the resulting powder was weighed. PCs was extracted from powder with 60% (v:v) ethanol at a ratio of 1:100 (wt/vol), with an ultrasonic power of 300 W for 30 min, and then centrifuged at 10,000×g for 10 min to obtain the supernatant, which was used for the analysis. Under acidic conditions, resorcinol and phloroglucinol on the A-ring of PCs condensed with vanillin to produce colored compounds with a characteristic absorption peak at 500 nm. The absorbance at 500 nm was detected by microplate reader. The PCs content in WM were calculated according to a standard curve and results obtained from three biological samples.

### Animals and sample collection

The animal study protocols were approved by the Animal Care and Use Committee of Lanzhou University (CY20190801). No lamb was harmed during the feeding trial.

In this study, 54 healthy Hu lambs at three months, with average body weight of 25.02±1.81 kg, were divided into three groups according to similar body weight and housed in individual pens (1.0 m×1.5 m). Ram lambs in control group (WM0, n = 18) were fed diets without WM, and two treatment groups were fed diets prepared with 4% (WM4 group, n = 18) and 8% (WM8 group, n = 18) of WM. Raw materials of mulberry feeds were obtained by crushing the above ground part and then mixed with other roughages to form a total mixed ration pellet. The composition and nutrient levels of experimental diets are shown in Table 1. The diet without WM was fed in the pre-feeding period for seven days, and the corresponding diet of the treatment group was fed in the formal period of 63 days. All ram lambs were fed at 08:00 and 16:00, with ad libitum access to food and fresh water. After the 70-day feeding trial, 15 ram lambs from each group were randomly selected and slaughtered for sample collection. On the 70th day, before morning feeding, jugular vein blood was collected from each sheep. The blood samples were centrifuged at 1,300×g for 15 minutes and the supernatant was collected and stored at −20°C. Testes and epididymides were weighed. Tissue samples from left testes were collected and placed in liquid nitrogen for further research. Mulberry planting and lamb raising were conducted at Minqin Defu Agriculture Co., Ltd. (Minqin, China).

### Measurement of triglyceride, total cholesterol, total antioxidant capacity, and malondialdehyde

Frozen testis sample was fully grounded into powder in liquid nitrogen, and then the tissue powder (100 mg) was mixed to 900 μL phosphate-buffered saline in a centrifuge tube, followed by vibration for 10 s, ice water bath for 30 s, and repeated two to three times. As for cells, about a million cells were gathered to prepare a cell homogenate by using ultrasonication. The homogenate was centrifuged at 13,000×g for 5 min at 4°C. Protein concentration was measured by a Bradford protein assay kit (Biosharp, Hefei, China). Triglyceride (TG), total cholesterol (T-CHO), total antioxidant capacity (T-AOC), and malondialdehyde (MDA) were determined according to the instructions of the kit provided by Nanjing Jiancheng Bioengineering Institute (Nanjing, China). The concentrations of TG, T-CHO, T-AOC, and MDA were converted into units per milligram or gram protein.

### Reverse transcription quantitative polymerase chain reaction analysis

The total RNA samples from the testis of the WM0, WM4, and WM8 groups were obtained by Trizol method. RNA quality was measured by agarose gel electrophoresis and Nanodrop 2000 spectrophotometer (Thermo Scientific, Wilmington, NC, USA). The qualified total RNA had an OD260/280 within 1.8–2.0 and an OD260/230 higher than 2.0. The cDNA was synthesized by utilizing the PerfectStart Uni RT with qPCR Kit (TransGen Biotech, Beijing, China). Eight genes related to steroidogenesis, apoptosis, and fatty acids metabolism were detected. The gene-specific primer sequences are presented in Table 2. Reverse transcription quantitative polymerase chain reaction (RT-qPCR) was performed using the CFX384 system (Bio-Rad, Hercules, CA, USA), and the reaction conditions were prepared as follows: initial denaturation at 95°C for 30 s, followed by 40 cycles at 95°C for 5 s and 61°C for 30 s. The relative expression levels of each gene were normalized to *hypoxanthine phosphoribosyltransferase 1* (*HPRT1*), *ribosomal protein S18* (*RPS18*), and *ribosomal protein lateral stalk subunit P2 (RPLP2*) by using 2^−ΔΔCt^ method.

### Cell culture and proanthocyanidins treatment

Immortalized sheep Leydig cell line and culture reagents used in this study were purchased from iCell Bioscience Inc. (Shanghai, China). PCs purchased from Solarbio (Beijing, China). Cells were cultured in Leydig cell immortalization specific culture medium supplemented with 10% fetal bovine serum and 100 IU/mL penicillin-streptomycin at 37°C in a humid atmosphere with 5% CO_2_.

### Cell proliferation assay

Leydig cells were seeded in 96 well plate at a density of 1×10^4^ cells/well. After 24 h of incubation, the cells were fully adhered to the wall and extended. The cells in the plate were divided into four groups (control [CK], PC5, PC10, PC20), and the final concentrations of PCs in each group were 0, 5 μM, 10 μM, and 20 μM. Each group was established 6 replicates, and cultured in 3 times repeatedly. Leydig cells were cultured for 12 h and then culture medium in the wells was discarded and 100 μL culture medium containing 10% CCK8 (Solarbio) was added to each well. After cultured for 1 h, the absorbance was detected at 450 nm.

### Necrosis and apoptosis detection

Necrotic and apoptotic cells were detected by using a YO-PRO-1/PI dual fluorescence detection kit in accordance with the manufacturer’s protocol (Beyotime, Shanghai, China). The green fluorescence emitted by YO-PRO-1 binding to DNA at 491 nm and the red fluorescence emitted by PI binding to DNA at 535 nm were observed through fluorescence microscopy.

### Enzyme-linked immunosorbent assay

The serum from the experimental animals and the culture medium from Leydig cell were collected to measure the level of testosterone (T) using a sheep T ELISA kit (YJ820701; Yuanju Tech, China) according to the manufacturer’s protocol. Plates were read on microplate reader at 450 nm. Each experiment was carried out in triplicate.

### RNA-Seq analysis

Total RNA was extracted from Leydig cells by using Trizol method. RNA integrity was assessed using the RNA Nano 6000 Assay Kit of the Bioanalyzer 2100 system (Agilent, Santa Clara, CA, USA). A total amount of 1 μg RNA per sample was used as input material for the RNA sample preparation. The library preparations were sequenced on an Illumina NovaSeq 6000 platform and 150 bp paired-end reads were generated by Biomarker Tech (Beijing, China). All downstream analyses were based on high-quality clean data (clean reads), which were obtained by removing reads containing adapter, poly-N and low-quality reads from raw data. Q20, Q30, and guanine-cytosine contents of the clean data were calculated. The paired-end clean reads were aligned to the *ovis aries* reference genome (Oar_v4.0, GCA_000298735.2) by using Hisat2 software [9]. Gene expression levels were estimated by calculating the fragments per kilobase of transcript sequence per million base pairs sequenced (FPKM). Differential expression analysis of the two groups was performed by DESeq2 R package (1.20.0). Differentially expressed genes (DEGs) were assigned as p-value<0.05 and Fold Change>1.5. All DEGs were used for Gene Ontology (GO) and Kyoto Encyclopedia of Genes and Genomes (KEGG) enrichment analysis through the ClusterProfiler R package. A term or pathway with p-value<0.05 was considered enriched.

### Statistical analysis

The experimental results were analyzed by IBM SPSS 19.0 (SPSS, Chicago, IL, USA) and were presented as the group mean±standard error of mean. Normal parameters were analyzed by Shapiro-Wilk test. The homogeneity of variance was analyzed by Levene’s test. Significant differences among three groups were analyzed by one-way ANOVA. Multiple comparisons between groups were compared with Tukey HSD. Significant differences between two groups were analyzed by Student’s *t*-test. P-value<0.05 was considered statistically significant.

## RESULTS

### Proanthocyanidins content in whole-plant mulberry

The correlation of standard curve was good, with a correlation coefficient (r) of 0.99. The PCs content in WM was 56.93±2.146 mg/g.

### T levels in the serum of rams

**Figure 1:**
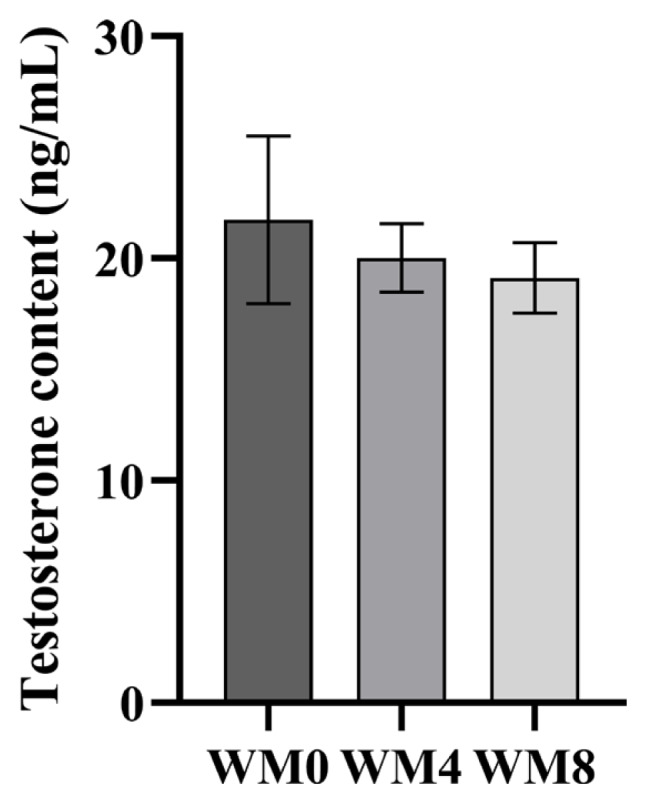
Levels of testosterone (T) in serum among different groups.WM0, without whole-plant mulberry; WM4, 4% whole-plant mulberry; WM8, 8% whole-plant mulberry.

The T levels in the serum of Hu sheep showed no significant differences among the three groups (Figure 1).

### Whole-plant mulberry stimulated the testicular development of rams

The addition of WM did not affect the body weight and epididymis weigh of Hu sheep (p>0.05, Table 3). The right testicular weight was significantly higher in the WM8 group compared to the WM4 and WM0 groups (p<0.05, Table 3). The total testicular weight (p = 0.06) and left testicular weight (p = 0.07) was elevated in the WM8 group compared to the WM0 group.

### Whole-plant mulberry increased total antioxidant capacity level and decreased total cholesterol content in the testis of rams

**Figure 2:**
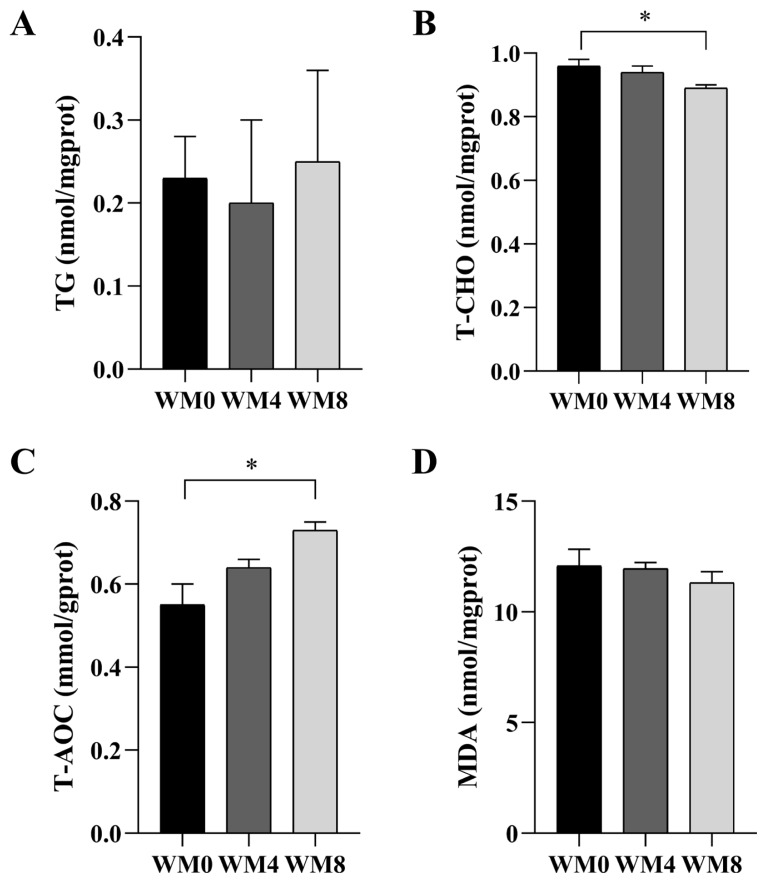
Levels of triglyceride (TG) (A), total cholesterol (T-CHO) (B), total antioxidant capacity (T-AOC) (C) and malondialdehyde (MDA) (D) in testes among different groups. * p<0.05. WM0, without whole-plant mulberry; WM4, 4% whole-plant mulberry; WM8, 8% whole-plant mulberry.

TG content was highest in the WM8 group, followed by the WM0 group, with no significant difference among the three groups (Figure 2A). T-CHO content decreased with the increase of WM concentration in the diet, and a significant difference was found between the WM8 and WM0 groups (p<0.05, Figure 2B). The T-AOC level in the WM8 group was significantly higher than that in the WM0 group (p<0.05, Figure 2C). There was no significant difference in MDA content with the increase of WM addition amount (p>0.05, Figure 2D).

### Gene expression in testis

**Figure 3:**
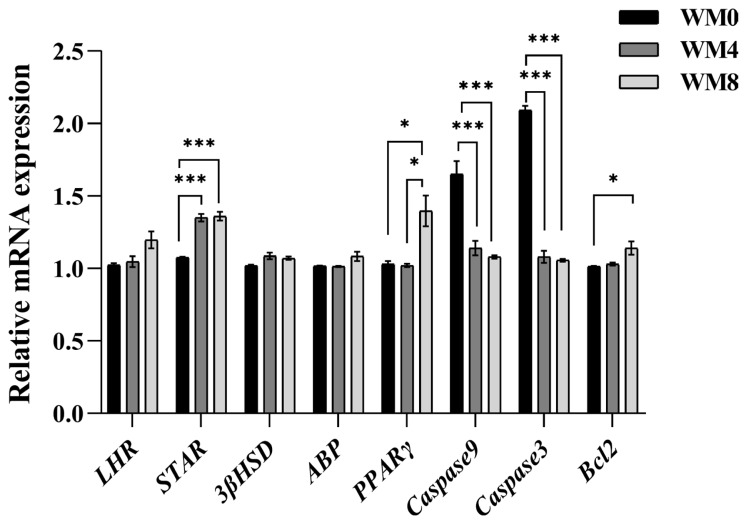
The expression levels of genes related to steroidogenesis, apoptosis, and fatty acids metabolism in the testis of rams. * p<0.05. ** p<0.01. *** p<0.001. WM0, without whole-plant mulberry; WM4, 4% whole-plant mulberry; WM8, 8% whole-plant mulberry. Values were shown as mean±standard error of the mean.

RT-qPCR analysis results indicated that *StAR* and *PPARγ* were significantly upregulated in the WM8 group (p<0.05, Figure 3). Genes related to apoptosis, such as *Caspase9* and *Caspase3* were significantly decreased (p<0.001) in the WM4 and WM8 groups compared to the WM0 group. The apoptosis suppressing gene, *Bcl2*, was significantly increased (p<0.05) in the WM8 group compared to the WM0 group. *LHR*, *ABP* and *3βHSD* showed no significant differences among the three groups (p>0.05).

### Proanthocyanidins promoted proliferation and suppressed necrosis of Leydig cells

**Figure 4:**
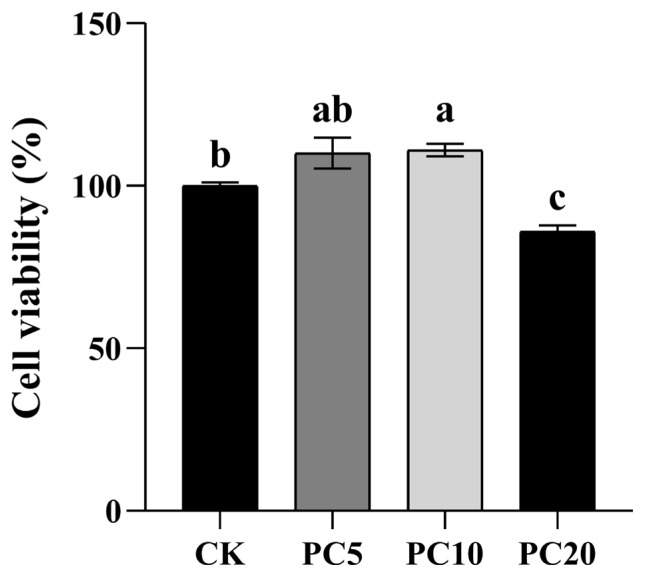
Cell viability of control (CK), 5 μM proanthocyanidins (PC5), 10 μM proanthocyanidins (PC10), and 20 μM proanthocyanidins groups (PC20). ^a–c^ Different letters indicate significant differences (p<0.05).

**Figure 5:**
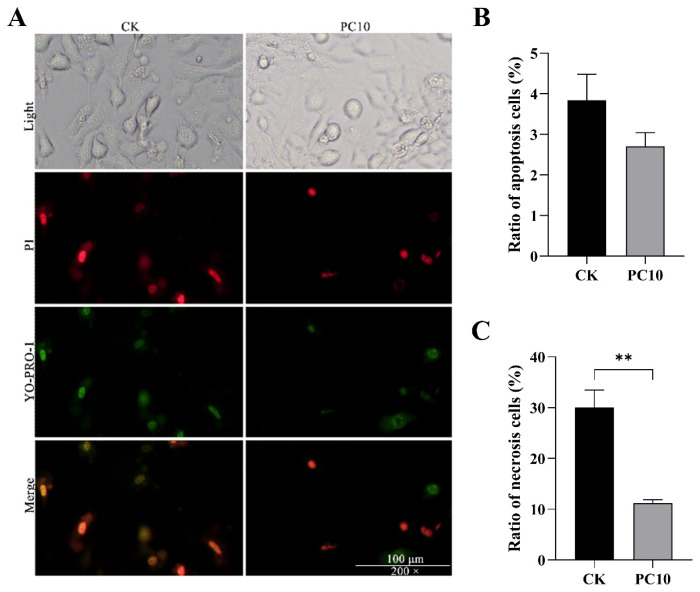
The apoptosis ratio and necrosis ratio of Leydig cells between the control (CK) and 10 μM proanthocyanidins (PC10) groups. (A) The nuclei of apoptotic cells were stained with YO-PRO-1 (green). The nuclei of necrotic cells were simultaneously stained with YO-PRO-1 and PI, with overlapping red and green fluorescence appearing orange yellow. (B-C) Ratio of apoptosis cells and necrosis cells. ** p<0.01. YO-PRO-1, oxazole yellow; PI, propidium iodide.

Compared to the CK group, supplementation of 10 μM PCs was significantly increased the Leydig cells viability, while 20 μM PCs decreased the cell viability (p<0.05, Figure 4). Thus, PC10 was selected for more experiment. The nucleus of apoptotic cells was stained with green fluorescence by YO-PRO-1. The nucleus of necrotic cells was simultaneously stained with YO-PRO-1 and PI, with overlapping red and green fluorescence appearing orange yellow (Figure 5A). After calculation, adding 10 μM PCs had a modest effect on reducing the apoptosis ratio (p>0.05, Figure 5B) and significantly reduced necrosis ratio in Leydig cells (p<0.05, Figure 5C).

### Proanthocyanidins improved the total antioxidant capacity and T levels in Leydig cells

**Figure 6:**
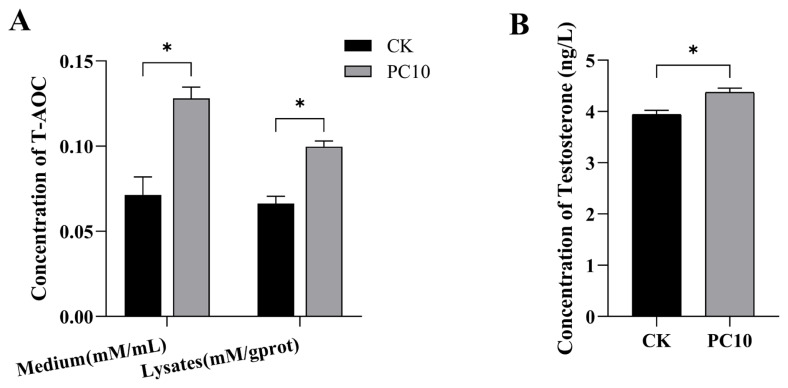
The concentration of T-AOC in the lysates and culture medium of Leydig cells (A) and testosterone in the culture medium (B) of control (CK) and 10 μM proanthocyanidins (PC10) groups. * p<0.05. T-AOC, total antioxidant capacity.

Compared to the CK group, adding 10 μM PCs significantly improved the T-AOC level in Leydig cells lysates and culture medium (p<0.05, Figure 6A). Meanwhile, T concentration in the culture medium was significantly elevated in the PC10 group (p<0.05, Figure 6B).

### RNA-sequencing and enrichment analysis of Leydig cells

**Figure 7:**
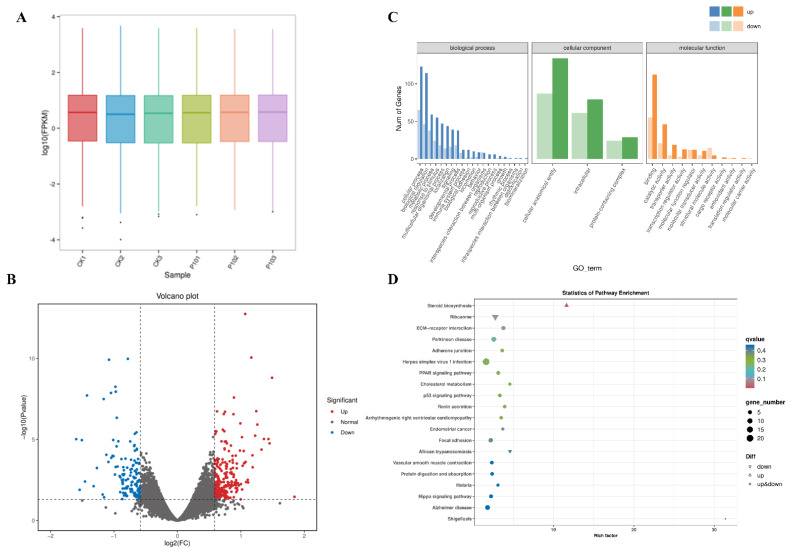
Differentially expressed genes (DEGs) identified in Leydig cells between the control (CK1, CK2, and CK3) and 10 μM proanthocyanidins (P101, P102, and P103) groups. (A) FPKM distribution. (B) Volcano plots of DEGs. The abscissa is the log2 (FoldChange) value, the ordinate is −log10 (p-value), and the grey dashed line indicates the threshold line of the differential gene screening criteria. Up-regulated genes are indicated by red dots and down-regulated ones by blue dots. (C) Gene ontology (GO) analysis of DEGs. (D) Kyoto Encyclopedia of Genes and Genomes (KEGG) enrichment analysis of DEGs. FPKM, Fragment Per Kilobase of transcript per Million mapped reads.

Transcriptome sequencing was performed on the CK (n = 3) and PC10 groups (n = 3). After quality control, 21,743,808 to 28,444,256 clean reads were obtained. The base mass value Q30 was over 91%, indicating a high sequencing quality. Approximately 95.58%–96.71% of the clean reads were mapped to the *Ovis aries* reference genome, and 89.82% to 90.52% were uniquely mapped (Table 4). The FPKM values of all samples were nearly identical (Figure 7A). After comparing the FPKM values between the CK and PC10 groups, 384 genes in the PC10 group were identified as DEGs according to p<0.05 and fold change>1.5, including 243 up-regulated genes and 141 down-regulated genes (Figure 7B). The GO analysis showed that the DEGs were significantly enriched in terms related to metabolic process and antioxidant activity (Figure 7C). Steroid biosynthesis pathway, p53 signaling pathway, cholesterol metabolism, PPAR signaling pathway, and Hippo signaling pathway was significantly enriched in the KEGG pathway enrichment analysis (p<0.05, Figure 7D; Table 5).

## DISCUSSION

As a widely planted and nutritious feed resource, mulberry leaves have been studied for their forage properties and nutritional value in different livestock, including sheep, cow calves, and pigs [10–12]. The supplementation of mulberry leaf to the diets of Hu sheep and beef cattle has improved the development of rumen papillae and stratum basale, nutrient digestibility, and rumen fermentation [13,14]. Silage mulberry leaves can also be used to feed steers, with no difference in growth performance and carcass quality compared to fresh mulberry leaves [15]. In the previously published data, the average daily gain of the WM8 group was significantly higher than that of the WM0 group. And there were no significant differences in final body weight, dry matter intake, and feed-to-weight ratio among the groups [16]. In the present study, the WM0, WM4, and WM8 groups were treated with WM instead of 0%, 20%, and 40% corn straw, respectively. The feed containing WM did not significantly differ from the WM0 group in dry matter, crude protein, neutral detergent fiber, and acid detergent fiber content. Thus, WM addition did not significantly affect Hu sheep body weight. However, adding 8% WM to the diet improved the testicular parameters.

ROS are involved in the regulation of cell apoptosis. Aitken et al [17] have demonstrated that exposure to electrophilic aldehydes, which are produced as a byproduct of sperm metabolism, triggers a prolonged apoptotic cascade in human spermatozoa. This cascade is initiated by an increase in mitochondrial ROS generation and culminates in oxidative DNA damage, DNA strand breakage, and cell death. Flavonoids, superoxide dismutase, polyphenols, and polysaccharides in mulberry leaves possess anti-aging and free radical scavenging effects [18]. Flavonoids and their metabolites may exert antioxidant property through various signaling pathways, such as PKC, MAPKs, and PI3K/Akt, which control cell survival [19]. In this study, we found that genes related to apoptosis, such as *Caspase9* and *Caspase3* were significantly downregulated. The apoptosis suppressing gene, *Bcl2*, was significantly upregulated in the WM8 group, indicating that dietary supplementation of WM could effectively inhibit cell apoptosis. Furthermore, adding 8% WM in the diet significantly improved the T-AOC level, indicating better oxidative and antioxidative balance in the testes of Hu sheep, which would be beneficial for spermatogenesis.

Previous research has shown that oxidative stress contributes to or causes the reduced T secretion that characterizes Leydig cell aging [20]. *In vivo* experiments have shown that feeding 1 g/kg/day mulberry leaf extract for two months can significantly reduce serum oxidative stress and increase the free T levels in diabetic rats [21]. Moreover, the addition of mulberry leaf flavonoids increased serum growth hormone, prolactin, and estradiol levels, and 45 g/day was the appropriate level to enhance milk performance and alleviate heat stress in buffaloes [22]. The synthesis of T initiates from the binding of luteinizing hormone (LH) to its receptor (LHR) on Leydig cells, subsequently activating the cAMP-PKA and SF-1 signaling pathways, which promotes the expression of the key enzyme STAR. As the precursor of T, cholesterol is transported to the inner mitochondrial membrane by STAR. Through the catalysis of P450scc, HSD3B1, CYP17A1 and HSD17B3, T is ultimately generated [23]. The synthesized T enters the lumen of the seminiferous tubules and binds to androgen-binding protein (ABP) secreted by Sertoli cells, forming a local high concentration of T within the seminiferous tubules, which stimulates the development of seminiferous tubules and spermatogenesis [24]. The T secretion could be modulated by LH, which occurs in a pulsatile manner. Therefore, the concentration of serum T would fluctuate to some extent, potentially introducing a certain bias into the assessment of serum T levels. However, in the present study, the gene expression level of *StAR* was significantly upregulated in the WM8 group, which would facilitate the T synthesis of WM8 group. Several findings support the hypolipidemic and hypotriglyceridemic effects of mulberry [18]. T can affect mitochondrial function, thus increasing energy metabolism and reducing fat levels [25]. However, the mechanism behind the observed lower T-CHO content in the testis of the WM8 group is not clear, which can be addressed in the future.

As natural antioxidants, PCs have an extremely strong antioxidant capacity. Research showed that PCs had a positive effect on steroid synthesis. Bashir et al [26] also found that grape seed-extracted PCs promoted steroid hormone synthesis and secretion through the PI3K/Akt/Nrf-2 signaling pathway. Currently, some studies have added grape seed PCs to semen preservation fluid to improve the viability of thawed porcine spermatozoa by reducing oxidative sperm damage and maintaining the structural and functional integrity of spermatozoa [27]. The results of the *in vitro* study confirmed our hypothesis that the addition of PCs increased the T-AOC level of Leydig cells, thereby reducing cell necrosis rates, and promoting T secretion. However, PC20 likely causes a decrease in cell viability due to its toxicity, which affects normal cell metabolism and function. While PC has a strong antioxidant capacity, moderate amounts of antioxidants can protect cells from oxidative stress. However, excessive amounts of antioxidants may disrupt the normal redox balance of the cell, thereby affecting the normal growth and division of the cell. Additionally, PC20 may influence cell cycle regulation, leading to cell arrest at a specific stage or inducing apoptosis, which in turn reduces cell viability. Using RNA sequencing technology, we found that changes occurred in the steroid biosynthesis pathway, p53 signaling pathway, cholesterol metabolism, PPAR signaling pathway, Hippo signaling pathway, PI3K/Akt signaling pathway, and oxidative phosphorylation in Leydig cells treated with 10 μM PCs. The genes enriched in the steroid biosynthesis pathway include *MSMO1*, *DHCR7*, *LSS*, *CYP24*, and *LOC101113583*, while *MSMO1*, *DHCR7* and *LSS* are involved in the enzymatic conversion of squalene to cholesterol. Genes enriched in the p53 signaling pathway include *MDM4*, *SESN2*, *LOC105610887*, *IGFBP3*, and *LOC101117527*. p53 is a tumor suppressor protein, which can protect normal cells from tumorigenesis due to DNA damage [28]. Research shows that, upon exposure to oxidative stress, p53 becomes activated to trigger apoptosis of TM3 cells [29]. As a key negative regulatory factor of p53, MDM4 can binds to p53 and inhibits its function, similar to MDM2 [30]. MDM4 participates in regulating cell proliferation, cell cycle arrest, and apoptosis processes [31]. SESN2 is involved in DNA repair and damage prevention (γ- or UV-irradiation, or doxorubicin) in a p53-dependent manner [32]. As a strong influencer on cell proliferation and a potent inhibitor of apoptosis, supplementing with exogenous IGF-1 can enhance survival, proliferation, and sperm production [33]. IGFBP3 can competitively bind to IGF1, thereby reducing the available binding of IGF1 molecules to IGF1R. In response to DNA-damaging ionizing radiation, p53 activates *IGFBP3* expression [34]. The downregulation of *IGFBP3* in the PC10 group indicates an enhanced inhibitory effect of IGF1 on cell apoptosis. The PPAR signaling pathway was also enriched in KEGG analysis. PPARγ is a nuclear fatty acid receptor, which can regulate the expression of downstream target genes by binding to specific ligands, such as fatty acids and carbohydrates, thus being involved in lipid metabolism and energy metabolism [35]. PPARγ affects glucose metabolism in human sperm through the pentose phosphate pathway. Moreover, the activation of PPARγ may induce capacitation and the acrosome reaction [36]. Many studies have shown that Hippo signaling pathway and PI3K/Akt signaling pathway participates in regulating multiple functions, such as cell apoptosis and proliferation [37]. PI3K/Akt and its downstream target protein mTOR jointly regulate the proliferation and differentiation of spermatogonial stem cells with multiple signaling molecules [38]. These results indicate that PCs may impact the biological pathways of T synthesis, lipid metabolism, cell proliferation, apoptosis, and antioxidation.

## CONCLUSION

The present study provides evidence that the addition of 8% WM to the diet can promote testis development, reduce T-CHO levels, and enhance antioxidant capacity in the testes of Hu sheep. *In vitro* tests on Leydig cells indicate that PCs alter the gene expression in steroid biosynthesis pathway, p53 signaling pathway, cholesterol metabolism, PPAR signaling pathway, and Hippo signaling pathway. These changes stimulate the antioxidant property, proliferation and T secretion of Leydig cells, which would be beneficial to spermatogenesis and testicular development.

## Figures and Tables

**Table 1:** Composition and nutrient levels of experimental diets for Hu sheep

Items	Groups^1)^		

	WM0	WM4	WM8
Ingredients (% of DM)	100.00	100.00	100.00
Corn straw	20.00	16.00	12.00
Mulberry	-	4.00	8.00
Corn bran	13.00	14.20	15.40
Corn	35.00	35.00	35.00
Corn germ meal	10.00	10.00	10.00
Cottonseed meal	1.00	1.00	-
Soybean meal	2.40	1.20	1.00
Molasses	2.00	2.00	2.00
NaCl	0.70	0.70	0.70
Protein supplementation	12.50	12.50	12.50
Per-mix	3.40	3.40	3.40
Nutritional levels (% of DM)			
Metabolizable energy (ME, MJ/kg)	8.46	8.64	8.71
Dry matter (DM)	88.85	86.82	88.11
Crude protein (CP)	16.88	17.13	16.90
Ether extract (EE)	2.51	2.49	2.57
Neutral detergent fiber (NDF)	46.76	46.53	45.78
Acid detergent fiber (ADF)	20.37	20.61	19.43
Acid detergent lignin (ADL)	3.84	4.57	5.42
Ca	0.93	0.90	0.87
P	0.29	0.28	0.26

1) WM0, without whole-plant mulberry; WM4, 4% whole-plant mulberry; WM8, 8% whole-plant mulberry.

**Table 2:** Primer sequences used for RT-qPCR

Gene	Primer sequence (5′-3′)
*ABP*	F: CCTGCCCTGTGTTTTATGGA
XM_012190148.2	R: CTATAGCCAGAGCAATCCTCA
*Bcl2*	F: CGCATCGTGGCCTTCTTT
XM_060405321.1	R: CGGTTCAGGTACTCGGTCATC
*Caspase3*	F: TCAGGGAAACCTTCACGAGC
XM_060406953.1	R: CCTCGGCAGGCCTGAATAAT
*Caspase9*	F: GCCAAGCCAAGGAAAACTCG
XM_060396599.1	R: CACGGCAGAAGTTCACGTTG
*HPRT1*	F: CGACTGGCTCGAGATGTGAT
XM_015105023.2	R: TCACCTGTTGACTGGTCGTT
*LHR*	F: AATGGCGGTCCTCATCTTCA
NM_001278566.1	R: ATACAGAAACGGATTGGCGC
*PPARγ*	F: ACGGCGGCTTCTACACCGACT
XM_015093026.1	R: ACGGCGGCTTCTACACCGACT
*RPLP2*	F: AGCGCCAAGGACATCAAAAAG
XM_004023349.4	R: TGGCCAGCTTGCCGATAC
*RPS18*	F: CACTGAGGACGAGGTGGAAC
XM_004018745.3	R: CTGTGGGCCCGAATCTTCTT
*StAR*	F: CCCAGCTGCGTGGATTTATC
NM_001009243.1	R: CTCTCCTTCTTCCAGCCCTC
*3βHSD*	F: GAATCGGCATGGTTCTGTCC
XM_012183658.1	R: CCGTAGATGTACATGGGCCT

RT-qPCR, reverse transcription quantitative polymerase chain reaction; *ABP*, androgen-binding protein; *Bcl2*, B-cell lymphoma-2; *HPRT1*, hypoxanthine phosphoribosyl transferase 1; *LHR*, luteinizing hormone receptor; *PPARγ*, peroxisome proliferators-activated receptors γ; *RPLP2*, ribosomal protein lateral stalk subunit P2; *RPS18*, ribosomal protein S18; *StAR*, steroid acute regulatory protein; *3βHSD*, 3β hydroxysteroid dehydrogenase.

**Table 3:** Descriptive statistics of reproductive traits among different groups

Testicular parameters^1)^	Mean±SEM		

	WM0^2)^	WM4	WM8
Initial body weight (kg)	24.64±0.51	24.41±0.56	24.72±0.59
Body weight before slaughter (kg)	41.36±0.47	42.00±0.57	43.30±0.73
Testicular index (g/kg)	5.41±0.66	5.38±0.55	7.08±0.44
Epididymal index (g/kg)	0.90±0.04	0.86±0.05	0.86±0.03
Total testicular weight (g)	224.13±28.23	225.93±22.95	306.06±18.72
Left testicular weight (g)	112.16±13.78	113.68±11.07	150.72±9.52
Left testicular length (mm)	80.27±4.03	80.67±2.78	86.67±1.28
Left testicular width (mm)	54.80±2.82	56.00±1.92	60.50±3.00
Right testicular weight (g)	111.98±14.52^b^	112.26±11.96^b^	155.33±9.30^a^
Right testicular length (mm)	79.93±3.72	80.44±3.65	86.25±1.75
Right testicular width (mm)	55.79±2.95	55.00±1.86	60.50±1.68
Total epididymal weight (g)	36.97±1.67	36.09±2.01	37.39±1.46
Left epididymal weight (g)	19.10±0.92	17.69±1.27	18.74±0.82
Right epididymal weight (g)	17.87±0.86	18.40±0.82	18.64±0.68

1) Testicular index, testicular weight/average body weight; Epididymal index, epididymal weight / average body weight.

2) WM0, without whole-plant mulberry; WM4, 4% whole-plant mulberry; WM8, 8% whole-plant mulberry.

a,b Values within a row with different superscripts differ significantly at p<0.05.

SEM, standard error of the mean.

**Table 4:** Quantity and quality of the sequencing data between the CK and PC10 groups of Leydig cells

Samples^1)^	Total reads	Clean reads	Clean bases	GC content (%)	Q30^2)^ (%)	Mapped reads (Ratio)	Uniquely mapped reads (Ratio)
CK1	43,487,616	21,743,808	6,504,043,180	50.19%	95.50%	42,054,880 (96.71%)	39,364,608 (90.52%)
CK2	49,468,310	24,734,155	7,398,638,376	51.12%	91.69%	47,323,740 (95.66%)	44,432,059 (89.82%)
CK3	51,032,746	25,516,373	7,631,106,600	50.49%	91.93%	48,856,774 (95.74%)	45,948,921 (90.04%)
P101	56,888,512	28,444,256	8,508,212,520	50.16%	95.27%	54,880,112 (96.47%)	51,471,540 (90.48%)
P102	46,323,626	23,161,813	6,928,561,952	50.41%	92.20%	44,329,676 (95.70%)	41,788,783 (90.21%)
P103	46,477,664	23,238,832	6,953,365,292	50.45%	91.45%	44,423,279 (95.58%)	41,930,439 (90.22%)

1) CK, control group (CK1, CK2, CK3). PC10, treated with 10 μM proanthocyanidins (P101, P102, P103).

2) Q30, the percentage of bases with a Phred quality score above 30%.

GC, guanine-cytosine.

**Table 5:** Kyoto Encyclopedia of Genes and Genomes (KEGG) enrichment analysis

KEGG_A_class	KEGG_B_class	ID	Pathway	p-value	Up-regulated genes	Down-regulated genes
Metabolism	Lipid metabolism	ko00100	Steroid biosynthesis	5.88E-05	*CYP24, DHCR7, LSS, MSMO1, LOC101113583*	
Cellular processes	Cell growth and death	ko04115	p53 signaling pathway	0.009308	*MDM4, SESN2, LOC105610887*	*IGFBP3, NewGene_3312, LOC101117527*
Organismal systems	Digestive system	ko04979	Cholesterol metabolism	0.011281	*LIPG, LRP2*	*ABCA1, ANGPTL4*
Organismal systems	Endocrine system	ko03320	PPAR signaling pathway	0.012293	*HMGCS1, OLR1, SCD, SORBS2*	*ANGPTL4, MMP1*
Environmental information processing	Signal transduction	ko04390	Hippo signaling pathway	0.038158	*AXIN2, CCN2, PPP2R2B, NewGene_3810, NewGene_3870*	*CTNNA3, WNT5B*
Environmental information processing	Signal transduction	ko04151	PI3K/Akt signaling pathway	0.072384	*COL4A6, FGF21, ITGA11, ITGB84, PPP2R2B, THBS2, LOC105610887, LOC11411300*	*COL9A2, LOC114112251, LOC114112675*
Metabolism	Energy metabolism	ko00190	Oxidative phosphorylation	0.089534		*NDUFA4L, LOC101107153, LOC101114379, LOC101121036, LOC114110252*
